# Changes in the diversity of ginseng endophyte flora driven by *Fusarium solani*

**DOI:** 10.3389/fmicb.2025.1554706

**Published:** 2025-04-28

**Authors:** Zhuo Sun, Yang Hu, Yi-Xin Yang, Meng-Yuan Lei, Zhong-Ming Han, Lin Cheng, Wan Wang, Mei Han, Ze-Liang Lyu, Li-Min Yang

**Affiliations:** ^1^College of Chinese Medicinal Materials, Jilin Agricultural University/Cultivation Base of State Key Laboratory for Ecological Restoration and Ecosystem Management of Jilin Province, Changchun, China; ^2^State Local Joint Engineering Research Center of Ginseng Breeding and Application, Changchun, China; ^3^Changchun Medical College, Changchun, China

**Keywords:** *Panax ginseng*, root rot, endophytic flora, taxonomic diversity, community structure

## Abstract

Endophytic flora serves a crucial function as a secondary line of defense against pathogen invasion in plants. To investigate the mechanisms underlying the relationship between changes in endophytic flora and ginseng root rot, exhumate beneficial endophytic bacteria, and explore biological management approaches for ginseng root rot. In this study, we used Illumina high-throughput sequencing and bioinformatics analysis to investigate the characteristics and differences in endophytic microbial community structure between healthy ginseng (HG) and diseased ginseng (BLS) after *Fusarium solani* infection. The findings revealed that as ginseng root rot increased, the diversity and richness of endophytic bacterial communities increased before decreasing, but the diversity and richness of endophytic fungal communities decreased. The dominating bacterial phylum in ginseng roots was *Proteobacteria*, which declined in quantity as the disease progressed. *Ascomycota* was the dominating fungal phylum among endophytes, and its prevalence grew as the disease progressed. At the genus level, the relative abundance of *Rhodococcus*, *Stenotrophomonas*, Var*iovorax*, and *Achromobacter* species increased with the occurrence of ginseng root rot, in contrast, *Pantoea* and *Pseudomonas* species decreased in relative abundance as the prevalence of ginseng root rot increased. The relative abundance of the pathogenic fungi *Gibberella*, *Nectria*, *Ilyonectria*, and *Alternaria* in ginseng roots increased as the disease progressed. Endophytic fungal LEfSe research revealed that *Neonectria* was the particular biomarker discovered in the highly susceptible group. Additionally, commensal nutrient-type fungi appeared to be absent in moderately susceptible ginseng, but pathognomic nutrient-type fungi grew, coupled with potentially pathogenic fungi, exacerbating the condition. These results suggest that there is a pattern of response of ginseng endophytic microbial diversity to disease infestation. In this work, we investigated the impact of varying degrees of root rot on ginseng’s endophytic flora structure. The study’s findings give a theoretical framework for understanding the microecological processes of ginseng root rot via the lens of microbial ecology and applying biological control tools.

## 1 Introduction

*Panax ginseng* C.A. Meyer is a perennial medicinal plant of the family Wujiaceae, which is an important medicinal plant resource in China and is widely distributed in China, South Korea, Russia, and other countries. Northeast China is the main producing area of ginseng, and ginseng cultivation has become one of the important pillar industries in the local area (Shen et al., 2015). At present, more than 40 types of ginseng diseases have been found, among them, ginseng root rot, caused by *Fusarium solani*, can cause softening and rotting of the roots, as well as green and yellow discoloration of the leaves, in severe circumstances, the entire ginseng plant may die (Farh et al., 2018; Wang et al., 2022), together with excessive soil moisture content (Zhao et al., 2014) and ginseng succession barriers (Tan et al., 2017), these factors contribute to a perennial morbidity rate of over 30% in three-year-old ginseng, and the mortality rate caused by root rot in six-year-old ginseng can reach 50 to 80%, significantly impacting the yield and quality of ginseng (Qi et al., 2022).

Endophytes are microorganisms that reside inside healthy plant tissues or organs, as well as in the intercellular spaces, but they do not cause disease in the host plant. With the advancement of endophyte research, it has been discovered that endophytes can not only inhibit plant diseases through various mechanisms such as competition, antagonism, and hyper-parasitism, but also have biological effects on the host plant, such as increasing stress resistance and regulating the accumulation of secondary metabolites (Giauque et al., 2019; Li et al., 2023; White et al., 2019). Furthermore, it has been suggested that endophytic flora is an important part of the host plant, and that it has formed a functional community with the host in a long-term co-evolutionary process that is mutually beneficial and under dynamic change (Wu et al., 2017; Christian et al., 2016).

Plant endophytic flora, like human intestinal flora, live in plant tissues and exchange materials and energy with the plant, playing an important role in plant growth, reproduction, and health (Chu and Bae, 2022; Huang et al., 2024). According to studies, the type, number, and metabolic activity of plant endophytes are critical to plant health as well as the prevention and control of soil-borne illnesses (Deng et al., 2015). Plant endophytes can interfere with harmful bacteria’s molecular mechanisms or compete for ecological niches. They can also operate as external influences, inducing plant resistance or activating the plant immune system via molecular pattern recognition (Ma et al., 2021). However, when the dynamic equilibrium of plant endophyte flora is disrupted by disease infestation, it affects the structural composition and abundance of the plant endophyte flora, leading to a pathological imbalance in the diversity of plant endophyte microorganisms. This imbalance may directly result in the development of the disease (Wang et al., 2021). Busby et al. (2016) thoroughly investigated the connections between the endophytic flora of *Fallopia japonica* and pathogenic bacteria. They discovered that the presence of endophytic fungi, such as *Phomopsis* spp. can alter the balance of endophytic flora variety in *Fallopia japonica* in combination with the pathogenic fungus Puccinia polygoni-amphibii var. Torvariae leads to the emergence of leaf rust. It is implied that pathogenic bacteria may induce ginseng disease by disrupting or destroying the diversity of ginseng endophyte flora. This transformation shifts the ginseng endophyte flora from a state of physiological equilibrium to a pathologic imbalance, worsening ginseng sickness or possibly leading to death. However, no studies have been conducted on the pathophysiology of ginseng illnesses in terms of endophytic flora balance. Therefore, by analyzing the structural characteristics and differences in microbial communities within the roots of ginseng at healthy and different disease levels, the present study investigated the relationship between the structural changes of ginseng endophytic flora and the occurrence of the disease, which is of great significance for research on biological control methods of ginseng root rot disease.

## 2 Materials and methods

### 2.1 Test strains and ginseng

The ginseng pathogenic fungus *Fusarium solani* was provided by the Department of Plant Pathology, College of Agriculture, Jilin Agricultural University, Jilin, China, and kept at 4°C for storage. The ginseng used was a 3-year old ginseng seedling, cultivar “Fuxing No.1,” provided by Jilin Ginseng King Plant Protection Technology Co.

### 2.2 Preparation of conidial suspension of pathogenic fungus

The activated *F. solani* was inoculated into the center of PDA medium (90 mm in diameter) and removed after 14 days of incubation at 25°C in a controlled environment incubator. The conidia on the surface of the medium were scraped off with an aseptic slide, collected in distilled water, agitated with sterile glass beads for 20 min, and then filtered through three layers of sterile gauze. Conidial suspensions approximately about 3 × 10^4^ conidia/mL prepared made in sterile deionized water and stored 4°C. Conidia counts were calculated using the hemocytometer counting method (Xie et al., 2023).

### 2.3 Field experiments and sample collection

The study on ginseng root rot susceptibility was done from June to August 2023 at the Garden of Medicinal Botany, Jilin Agricultural University, Changchun, Jilin Province, China (43°48′40″N, 125°25′1″E, 215 m). The new woodland soil was extracted from Fusong, China (42°23′36″N, 127°49′18″E, 420 m). The baseline values of the soil were 22.77 g/kg of organic matter, 304.21 mg/kg of alkaline dissolved nitrogen (ADN), 9.49 mg/kg of active phosphorus (AP), 189.67 mg/kg of fast-acting potassium (FAP), PH 6.42, and electrical conductivity (EC) 335.62 dS/m. The ginseng nursery soil was a blend of new forest soil and vermiculite in a 2:1 ratio. Take 3-year-old ginseng seedlings with compact growth and a well-developed root system. Sterilize the surface of ginseng roots with 20% sodium hypochlorite, rinse with sterile water (Sun et al., 2019), and transplant into polypropylene (PP) plastic pots (26 cm diameter, 18 cm height) with three plants per pot. Place the pots in a plastic greenhouse that receives 16 h of sunshine at 30°C and 8 h of darkness at 15°C. Keep the seedlings watered and fertilized on a regular basis. The susceptibility test was carried out by wounded root perfusion method after seedling establishment (Li et al., 1983). Each ginseng plant in the susceptible treatment group was infused with 50 mL of *F. solani* conidial suspension at a concentration of 3 × 10^4^ conidia/mL, whereas each ginseng plant in the healthy treatment group was injected with an equivalent amount of sterile water. Each treatment was replicated 30 times.

**Figure 1:**
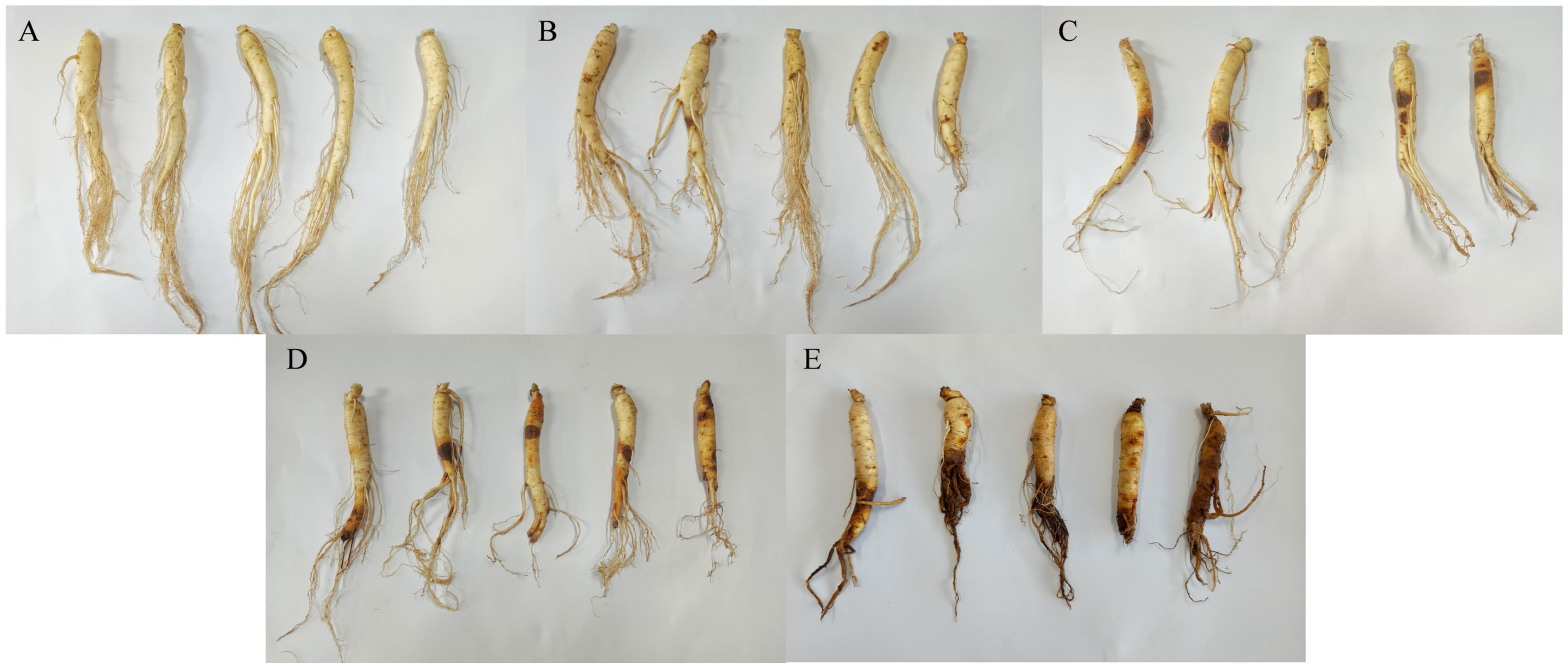
Characterize ginseng with different disease levels based on the percentage of lesion area. **(A)** HG, healthy ginseng; **(B)** BLS1, lesion area less than 20%; **(C)** BLS2, white mycelium appears in the root and the lesion accounts for 21–40% of the root surface area; **(D)** BLS3, lesion area 41–65% and severely affected appearance; **(E)** BLS4, lesion area greater than 66% or completely decomposed.

The degree of ginseng root rot disease was categorized into four levels according to the method described by Ma et al. (1983), namely: grade 0: healthy ginseng (HG); grade 1: lesions covering less than 20% of the root surface area (BLS1); grade 2: white mycelium appearing on the roots with lesions covering 21–40% of the root surface area (BLS2); grade 3: lesions covering 41–65% of the root surface area, with severely affected appearance (BLS3); grade 4: lesions covering more than 66% of the root surface area or completely rotted (BLS4). Here, the root area statistics include taproots and fibrous roots. Ginseng disease grades were investigated, and samples were collected 30 d after *F. solani* inoculation. Five ginseng samples were collected from each grade, totalling 25 samples (25 *P. ginseng* plants), which were individually placed in self-sealing bags and transported to the laboratory in an ice box (Figure 1).

### 2.4 Sterilization of ginseng roots

Sterilization of ginseng tissue components was optimized using the method described by Li et al. (2024) method. The soil on the ginseng root surface was shaken off and rinsed with sterile water to remove impurities. The ginseng root samples were cut into 0.5 ~ 0.8 cm pieces, and 2–5 g of ginseng root were placed in a beaker. The samples were soaked in 70% ethanol for 40 s and then in 2.5% sodium hypochlorite for 10 min. Subsequently, they were washed with sterile water five times and drained of any residual water. The ginseng tissue was frozen in liquid nitrogen for 1 h and then stored at −80°C.

### 2.5 DNA extraction, PCR amplification, and Illumina novaseq sequencing

Total microbiome DNA was extracted from each sample by the CTAB method, and the quality of DNA extraction was detected by agarose gel electrophoresis, while DNA was quantified by UV spectrophotometer (wavelength 260 nm). For the V3-V4 region of 16S rDNA, we chose the following primers to amplify the sequence in three rounds (Walters et al., 2016): fM1 (5’-CCGCGTGNRBGAHGAAGGYYYT-3′)-rC5 (5’-TAATCCTGTTTGCTCCCCAC-3′), fM1 (5’-CCGCGTGNRBGAHGAAGGYYYT-3′)-V4-rC5(5’-GACTACHVGGGTWTCTAATCCTGTTTGCTC-3′) and 515F (5’-GTGYCAGCMGCCGCGGTAA-3′)-806R (5′- GGACTACNVGGGTWTCTAAT-3′). For the ITS, two rounds of amplification of the sequence were performed using the following primers (Yao et al., 2019): ITS1F (5’-CTTGGTCATTTAGAGGAAGTAA-3′)-ITS4 (5’-TCCTCCGCTTATTGATATATGC-3′) and fITS7 (5′- GTGARTCATCGAATCTTTG-3′)-ITS4 (5’-TCCTCCGCTTATTGATATGC-3′). PCR amplification was performed using a 50 μL PCR amplification system: a 25 μL reaction system containing 25 ng of template DNA, 12.5 μL of PCR premix, 2.5 μL of each primer, and a regulated volume of PCR-grade water. Specific conditions detailing the endophytic fungal and endophytic bacterial PCR reactions are described in Supplementary Tables S1–S5. PCR products were confirmed by 2% agarose gel electrophoresis. Ultrapure water was used throughout the DNA extraction process to ensure the accuracy of the PCR results, and the PCR products were purified using AMPure XT beads (Beckman Coulter Genomics, Danvers, MA, United States) and then quantified using Qubit (Invitrogen, United States) Quantification. Finally, all samples were sequenced on the Illumina NovaSeq platform according to the manufacturer’s recommendations (sequencing service provided by LC-Bio, Hangzhou, China).

### 2.6 Data analyses

We utilized the ASV (amplicon sequence variation) feature sequence and abundance tables to conduct alpha and beta diversity studies. To assess the species richness and diversity of endophytic microbial communities, five indices were chosen to determine the alpha diversity of microorganisms: the number of observed ASVs, the coverage index, the Chao1 index, the Simpson index, and the Shannon index. Beta diversity was used to compare the microbial community composition among samples. Non-metric multidimensional scaling (NMDS) studies using the Bray-Curtis distance were conducted to examine inter- and intra-group relationships. The Wilcoxon rank-sum test (*p <* 0.05 is considered statistically significant) was used to analyze differences in alpha and beta diversity among multiple groups. In addition, to determine whether the differences between groups were significantly greater than the differences within groups, ANOSIM analysis was performed in the R software using the ANOSIM function of the vegan package. All the indices in our samples were calculated using QIIME and visualized with R software. Pearson’s correlation was used to evaluate the relationship between the relative abundance of microorganisms and the illness index (*p* < 0.05 is considered statistically significant and *p* < 0.01 is considered highly significant). Stacked plots categorize the relative abundance of TOP20 species, representing the relative abundance of each sample in a different form. The LEfSe analysis was performed to identify differentially abundant taxa to discover statistically significant biomarkers. LDA scores higher than 4 (endophytic bacteria) and greater than 3 (endophytic fungus) were considered as potential indicators. Microbial functional profiles were examined using the PICRUSt2 database,^1^ which predicts bacterial functional profiles, and the FUNGuild database.^2^ The STAMP software and the Wilcoxon rank-sum test (*p <* 0.05 is considered statistically significant) were then used to compare bacterial functional features in healthy and highly susceptible ginseng (Nguyen et al., 2016). All data have been uploaded to the NCBI database under the registration numbers PRJNA1167876 Bacteria (https://dataview.ncbi.nlm.nih.gov/object/PRJNA1167876?reviewer=gelpl7tjjacot9a9k2h3e36f59) and PRJNA1167695 fungi (https://dataview.ncbi.nlm.nih.gov/object/PRJNA1167695?reviewer=s1mkchug8c2qmglvaf5k9bdqpt).

## 3 Results

### 3.1 Sequencing and in-depth analysis

High-throughput sequencing of bacterial 16S rRNA yielded 1,748,777 valid sequences, accounting for 8,370 ASVs in total. High-throughput sequencing of the fungal internal transcribed spacer region (ITS) yielded 2,042,356 valid sequences, corresponding to 258 ASVs. Statistical analysis of the Coverage Sequencing Depth Index revealed that the coverage rate was close to 99% for each sample, indicating that all species information was fully detected. The sequencing data might accurately depict the structure and diversity of fungi and bacteria in each sample. Additional sequencing data would provide only a small number of novel species (ASVs; Supplementary Table S6).

### 3.2 Diversity and structure of endophytic microbial communities

**Figure 2:**
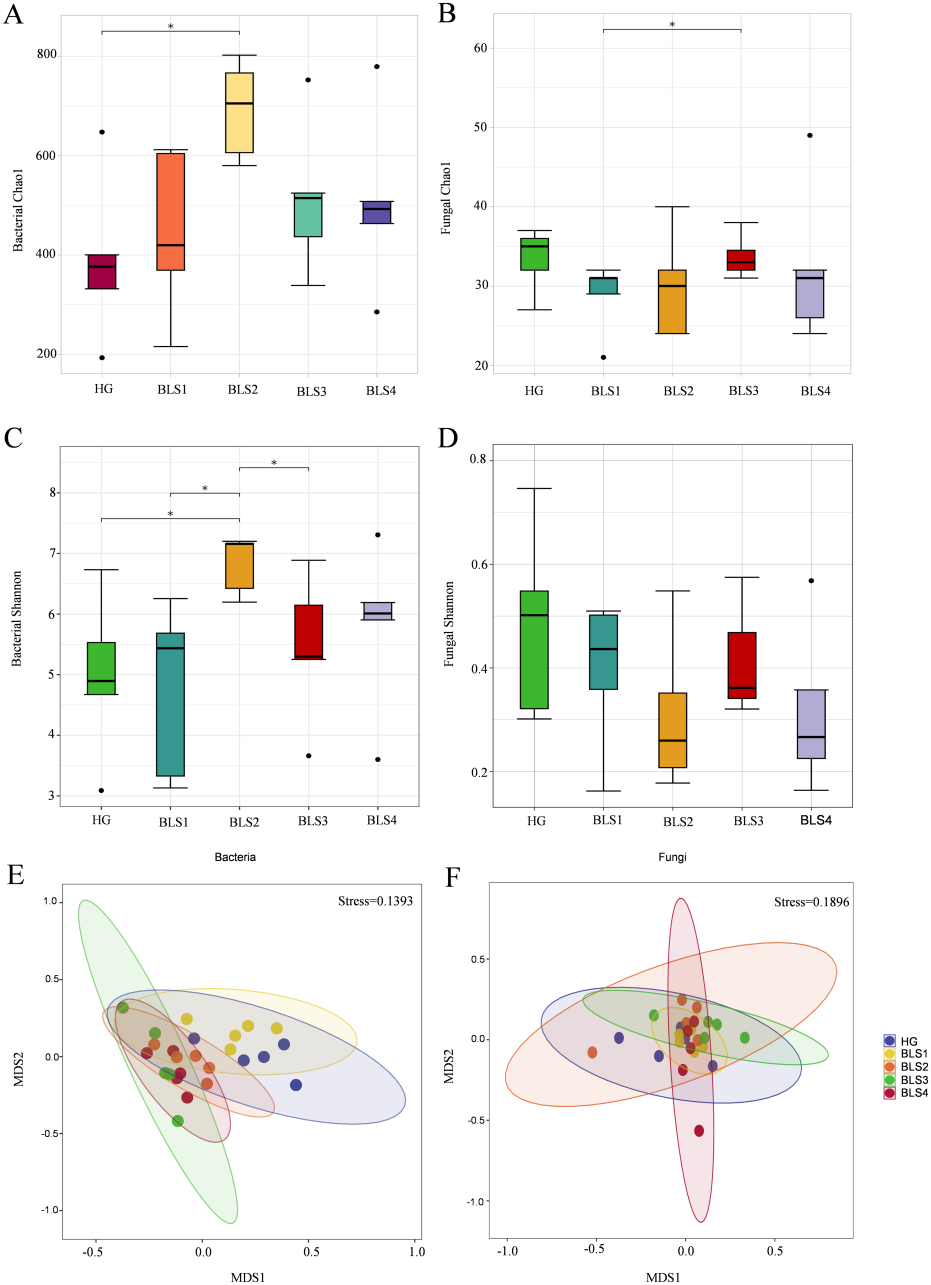
Changes in *α*-diversity and *β*-diversity of endophytic microorganisms in ginseng under different levels of root rot disease. **(A)** Chao 1 for endophytic bacteria, **(B)** Chao 1 for endophytic fungi, **(C)** Shannon for endophytic bacteria, **(D)** Shannon for endophytic fungi, **(E)** endophytic bacteria and **(F)** endophytic fungi. Data are presented as the mean ± standard error (*p* <  0.05, according to the Wilcoxon rank-sum test).

In the 16S rRNA-based analysis of endophytic bacteria, the number of operational taxonomic units (ASVs) and Chao1 index were significantly higher in the BLS2 group compared to the HG group (*p <* 0.05; Figure 2A; Supplementary Table S6). The Shannon index was significantly higher in the BLS2 group compared to HG, BLS1, and BLS3 (*p <* 0.05; Figure 2C). The BLS2 group had a significantly higher Simpson’s index compared to the HG and BLS1 groups (*p <* 0.05; Supplementary Table S6). ANOSIM analysis revealed significant differences between samples across all five groups (*R >* 0, *p <* 0.05). In addition, NMDS analysis using the Bray-Curtis distance was employed to analyze the diversity of endophytic bacteria in ginseng with varying levels of illness. The results indicated that the healthy ginseng HG and BLS1 groups were more similar. Meanwhile, the BLS2, BLS3, and BLS4 groups gathered together (Figure 2E).

Fungal ITS rRNA endophyte analysis revealed that the BLS3 group had a significantly higher Chao1 index compared to the BLS1 group (*p <* 0.05; Figure 2B). There were no significant differences in the number of ASVs, Shannon index, or Simpson index among the other five groups. However, the Shannon and Chao1 indexes of the BLS1 group, BLS2 group, BLS3 group, and BLS4 group were all lower than those of the HG group, with no significant difference (Figures 2B,D). The ANOSIM analysis revealed that the dissimilarities between samples in all five groups were greater than those within groups (*R >* 0), although these differences were not statistically significant. By using NMDS analysis of Jaccard distance, all five groups of samples clustered closely together with no significant separation (Figure 2F).

### 3.3 Composition and variation of bacteria within ginseng roots

**Figure 3:**
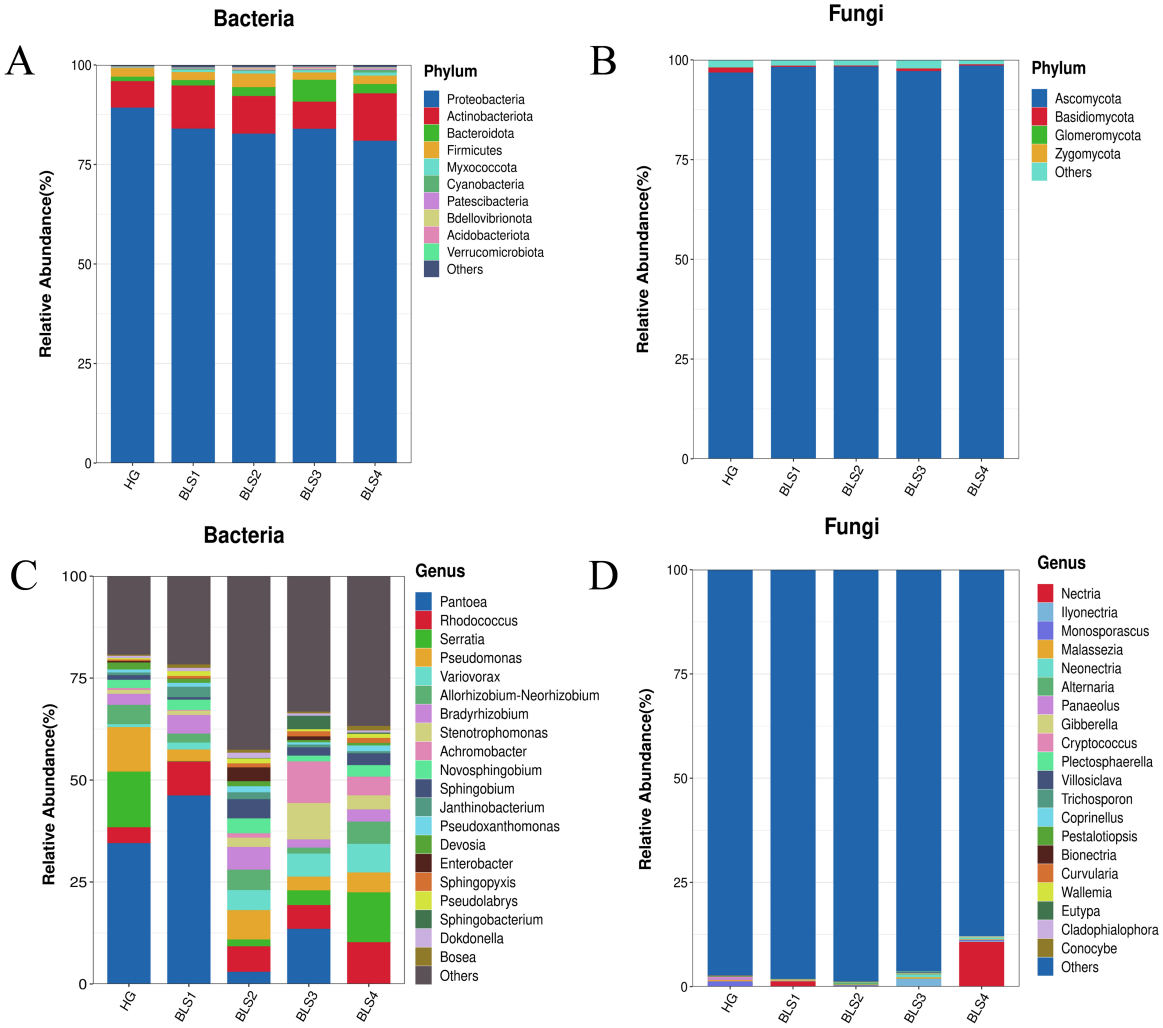
The composition and changes of endophytic microorganisms in healthy ginseng plants and ginseng with varying degrees of disease. **(A,C)** Endophytic bacteria at the phylum and genus levels, respectively, **(B,D)** endophytic fungi at the phylum and genus levels, respectively.

**Figure 4:**
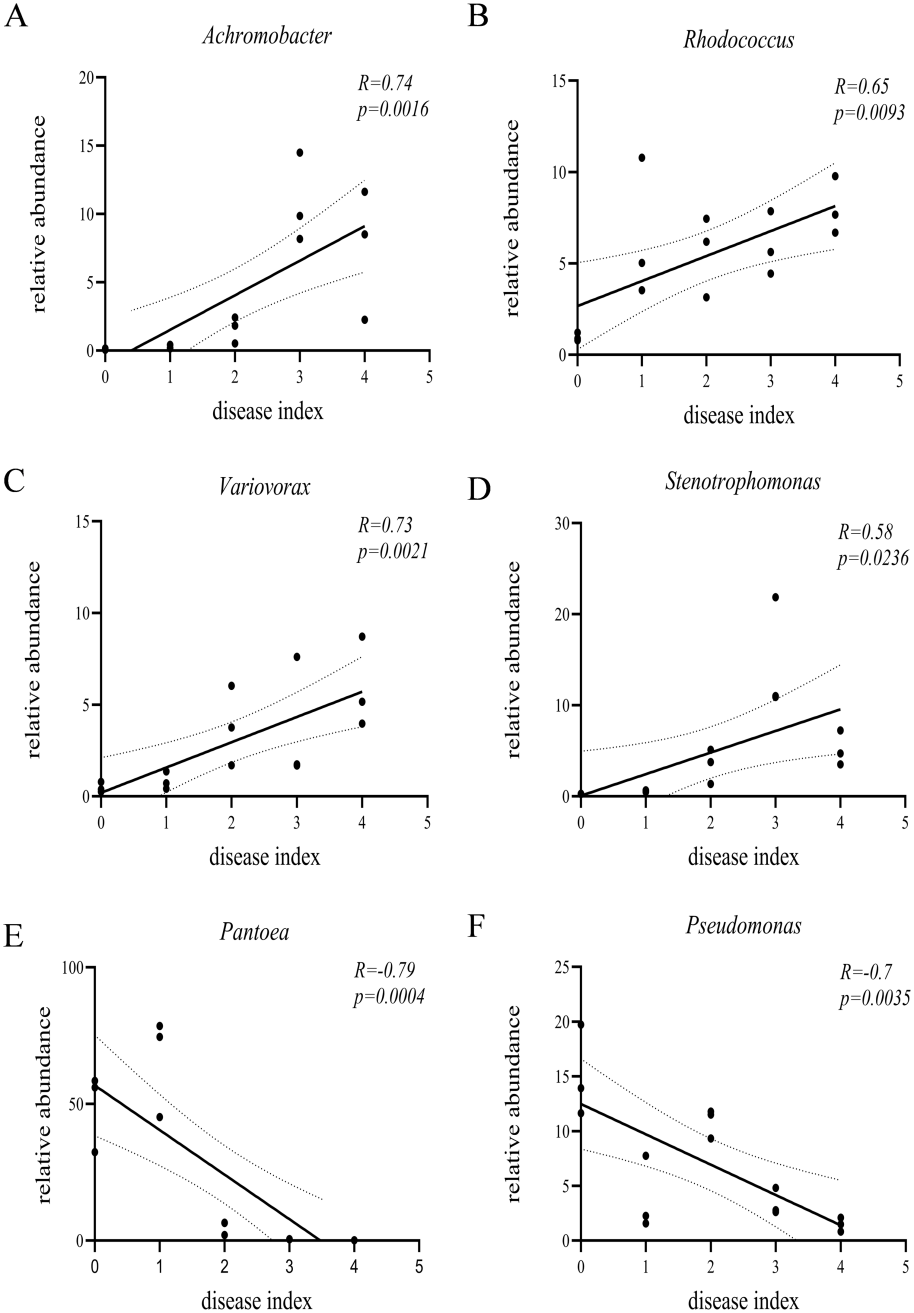
Pearson’s correlation analysis between relative abundance of specific bacterial genera **(A)** *Achromobacter*, **(B)** *Rhodococcus*, **(C)** *Variovorax*, **(D)** *Stenotrophomonas*, **(E)** *Pantoea*, and **(F)** *Pseudomonas* in ginseng roots and ginseng disease index.

Endophytic bacteria from ginseng roots were classified as ASV sequences with 100% similarity. The similar bacteria detected in separate samples were combined, revealing a total of 34 phyla, 101 orders, 227 families, 413 genera, and 928 species. The major phyla in the five sets of samples were *Proteobacteria*, *Actinobacterioteria*, *Bacteroidota*, and *Firmicutes* (Figure 3). *Proteobacteria* were the most abundant, and their relative abundance declined by 5.26, 6.54, 5.31, and 7.52%, respectively, as the disease progressed, compared to healthy ginseng. *Actinobacteriota* (with relative abundance increases of 4.81, 2.81, 0.16, and 5.27%, respectively) and *Bacteroidota* (with relative abundance increases of 0.23, 1.09, 4.37, and 1.19%, respectively) increased with disease progression compared to the HG group, while *Firmicutes* in BLS2 had the highest relative abundance (3.43%; Figure 3A). The genus-level analysis revealed that the composition of the endophytic bacterial communities varied among the five sample groups. *Pantoea* was the first dominating genus in both BLS1 and BLS3 (13.65–46.36%), the first dominant genus in BLS2 was *Pseudomonas* (7.12%) and the first dominant genus in BLS4 was *Serratia* (12.21%; Figure 3C). The correlation between the relative abundance of bacteria and the degree of ginseng disease revealed that the relative abundance of *Rhodococcus* (*p* < 0.01), *Stenotrophomonas* (*p* < 0.05), Var*iovorax* (*p* < 0.01), and *Achromobacter* (*p* < 0.01), all significantly enriched in the roots of diseased plants, was positively correlated with disease severity. On the contrary, the relative abundance of the genera *Pantoea* (*p* < 0.01) and *Pseudomonas* (*p* < 0.05), which were significantly enriched in the roots of healthy plants, correlated negatively with the degree of ginseng disease severity (Figure 4).

### 3.4 Composition and variation of fungi within ginseng roots

The ITS sequences of ginseng root fungi revealed 5 phyla, 16 orders, 31 families, 49 genera, 67 species, and 97 strains. In the phylum-level study, the dominant phyla of the five sample groups were *Ascomycota* and *Basidiomycota*. The relative abundance of *Ascomycota* increased with the severity of the disease by 1.42, 1.51, 0.38, and 1.74%, respectively, compared to the HG group. (Figure 3B). In a genus-level analysis, *Monosporascus* was the dominant genus in the HG and BLS2 groups, *Nectria* in the BLS1 and BLS4 groups, and *Ilyonectria* in the BLS3 group. *Nectria* and *Ilyonectria* (0.01%) were exceedingly rare or even absent in the BLS2 group but highly enriched in the BLS3 and BLS4 groups, with relative abundances of 10.86 and 2%, respectively. The relative abundance of *Gibberella* increased with the severity of the ginseng condition by 0.07, 0.02, 0.19, and 0.12%, respectively, compared to the HG group. The relative abundance of *Neonectria* and *Alternaria* was negligible in the HG group, but they increased as the disease progressed and reached the highest relative abundance in the BLS3 group, at 0.62 and 0.46%, respectively, (Figure 3D).

### 3.5 Biomarkers and varying degrees of taxon change

**Figure 5:**
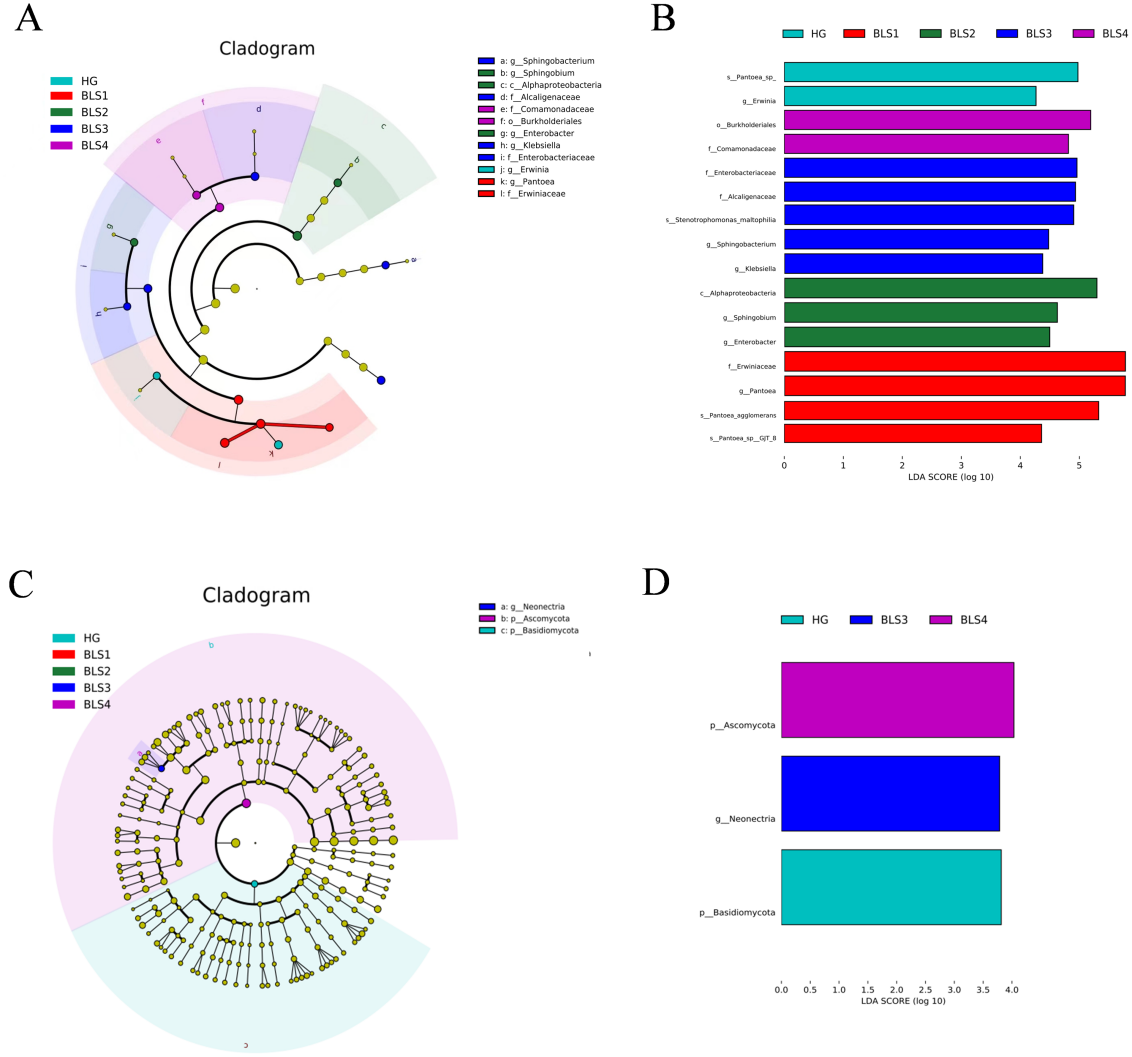
The linear discriminant analysis (LDA) effect size (LEfSe) analysis. **(A)** Diagram of endophytic bacterial differential taxa, **(B)** LDA scores of endophytic bacteria differential taxa (LDA score > 4), **(C)** diagram of endophytic fungi differential taxa. **(D)** LDA scores of endophytic fungi differential taxa (LDA score > 3).

We identified four bacterial taxa in the HG group with LDA scores greater than 4, such as *Erwinia* and *Pantoea*, through linear discriminant analysis (LDA) effect size (LEfSe). Four bacterial taxa in the BLS1 group had significantly high LDA (LDA > 4), including Erwiniaceae and *Pantoea*. In the BLS2 group, *Enterobacter*, *Sphingobium*, and Alphaproteobacteria had an LDA score greater than 4. *Klebsiella*, *Sphingobacterium*, and *Stenotrophomonas maltophilia*, as well as Alcaligenaceae and Enterobacteriaceae, had LDA scores exceeding 4, with *Enterobacteriaceae* exerting the most significant influence. Comamonadaceae and Burkholderiales were the only BSL4 groups with LDA scores greater than 4 (Figures 5A,B). Fungal LEfSe analysis revealed that *Basidiomycota* in the HG group all had LDA scores greater than 3. In the BSL4 group, *Ascomycota* had an LDA score greater than three. *Neonectria* exhibited a higher LDA score in the BLS3 group (Figures 5C,D).

### 3.6 Prediction of biological functions of endophytic microorganisms

**Figure 6:**
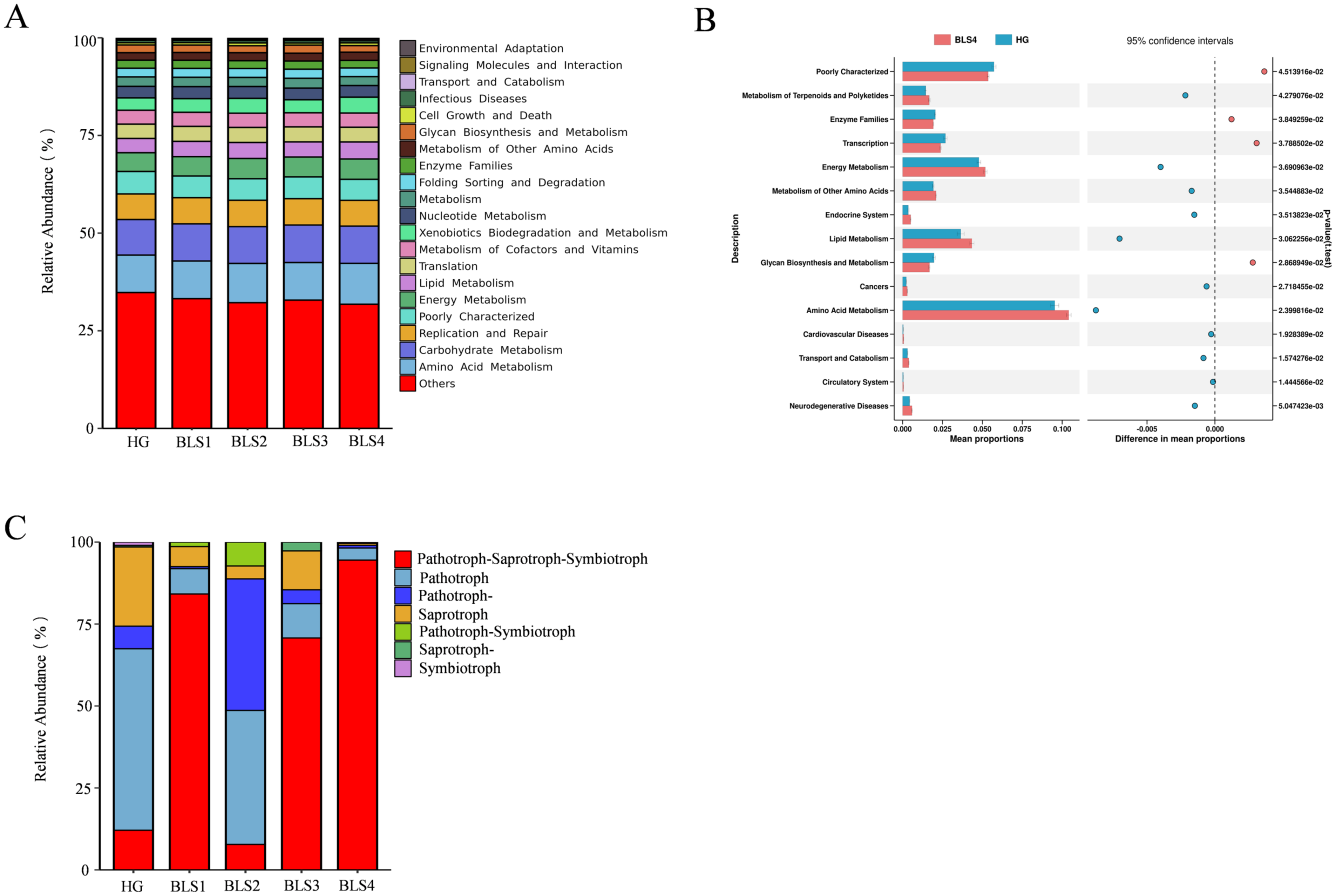
PICRUSt and FUNGuild were used to predict the ecological functions of bacteria and fungi in ginseng roots, respectively. **(A)** Secondary function analysis of KEGG bacteria from all samples; **(B)** Differences in KEGG secondary functions of bacteria in the roots of HG and BLS4 groups. We utilized graphical chapter analysis to illustrate variances at *p* < 0.05, and Welch’s t-test to establish significance. **(C)** Trophic phenotypes of fungal communities were found in all root samples.

Changes in microbial communities may affect the overall metabolic function of endophytic microbial communities. Ecological function prediction of bacteria and fungi in roots was conducted using PICRUSt and FUNGuild, respectively, to gain insights into microbial functions in healthy ginseng (HG) and all groups susceptible to ginseng (BLS; Figure 6). Secondary functional analysis of the KEGG pathway using PICRUSt2 to assess gene function in the microbial community revealed highly similar functional taxa and distributions in all groups. All groups exhibited enrichment in amino acid metabolism (relative abundance 9.57–10.46%), carbohydrate metabolism (9.11–9.59%), replication and repair (6.55–6.76%) and translation (3.66–3.85%), among others, totalling 15 subfunctions representing more than 1.0% (Figure 6A). The healthy ginseng group (HG group) and the severely susceptible group (BLS4 group) underwent STAMP difference analysis. The number of functions enriched in the severely susceptible group (BLS4 group) was significantly higher than in the healthy ginseng group (HG group). For instance, 11 functions, including amino acid metabolism, energy metabolism and lipid metabolism, were notably enriched in the severely susceptible group (BLS4 group). On the other hand, glycan biosynthesis and metabolism, transcription, and enzyme families were significantly enriched in the healthy ginseng group (HG group; Figure 6B).

The trophic functional types of endophytic fungi in ginseng samples were investigated using Funguild. The results revealed that Pathotroph-Saprotroph-Symbiotroph dominated the trophic types in the BLS1, BLS3, and BLS4 groups, with significantly higher levels than in the HG and BLS2 groups. Pathotroph-Saprotroph-Pathotroph were prevalent in the BLS2 group, with considerably larger abundances than in the HG group. Saprotrophs were the most abundant in the HG group, but they decreased significantly or disappeared after the ginseng illness (Figure 6C).

## 4 Discussion

### 4.1 Effect of disease occurrence on the diversity of microbial communities within ginseng roots

Microbial diversity and balance are critical to plant health (Yan et al., 2017). Endophytic flora’s resistance to pathogen invasion is strongly correlated with their diversity and richness (Wei et al., 2019). The moderately susceptible group (BLS2 group) had a significantly higher diversity of endophytic bacteria than the healthy group (HG group) based on Shannon and Simpson indices. We also discovered that the moderately susceptible group (BLS2 group) had a significant richness of endophytic bacteria community than the healthy group (HG group) based on the number of ASVs and the Chao1 index. This finding appears to indicate that the BLS2 group (diseased patches occupying 21–40% of the surface area of the ginseng root) is a cause for concern. This could be because when the ginseng disease level reaches the second level, the ginseng root becomes enriched with beneficial microflora to occupy ecological niches in response to the invasion of pathogenic bacteria, resulting in an increase in both bacterial diversity and richness in the moderately susceptible group (BLS2 group). Similar to the results of this investigation, Dai et al. (2022) showed that the bacterial community richness was higher in the root samples of strawberries with root rot than in the healthy samples (Dai et al., 2022). We used ANOSIM and NMDS analyses to better capture the differences in microbial community structure across and within groups. In the later phases of development (from BLS2 to BLS4 groups), there was a larger structural similarity in endophytic bacterial community diversity among the groups, confirming the similarity of endophytic bacterial communities in ginseng from distinct susceptibility groups. For fungus, the susceptible group (BLS) had a lower Shannon index and Chao1 index than the healthy group (HG), although there was no significant difference. This finding implies that root rot fungi impact ginseng, resulting in a decline in endophytic fungal population variety and richness. The NMDS analysis of endophytic fungi revealed no significant isolation of each sample, most likely because endophytic fungi do not form as ginseng illness progresses. To summarize, the beginning of ginseng root rot affects the diversity of microbial communities within ginseng roots, and the emergence and severity of ginseng root rot are linked to changes in the diversity of microbial communities within ginseng roots.

### 4.2 Effect of disease occurrence on the structure of microbial communities within ginseng roots

Determining the key endophytic microbial populations and their variations in ginseng roots is an important step in investigating the etiology of root rot disease. The *Proteobacteria* was the most dominant phylum among the endophytic bacteria in the five groups of samples, which is consistent with previous studies (Dasgupta et al., 2020). The abundance of *Proteobacteria* in healthy ginseng roots was higher than that in each diseased group (BLS). The abundances of *Actinobacteriota* and *Bacteroidota* increased with the aggravation of the disease. It has been reported that the phylum *Proteobacteria* is widely used in aspects such as the prevention and control of plant diseases and pests, as well as biological nitrogen fixation. In the endophytic fungal community, Ascomycota and *Basidiomycota* are the absolutely dominant phyla, both of which are common plant endophytes. Xie *et al*. analyzed the community change characteristics of fungi in the rhizosphere soil of garlic plants during the occurrence of garlic root rot. They believed that a high level of *Ascomycota* flora is closely related to the occurrence of garlic root rot (Xie et al., 2020). This study found that with the aggravation of ginseng diseases, the relative abundance of *Ascomycota* gradually increases, while the abundance of *Basidiomycota* decreases, which is consistent with the results of previous studies. This indicates that the secretions in the roots of healthy ginseng can inhibit the reproduction of *Ascomycota* and promote that of *Basidiomycota*. However, after ginseng is infected with root rot, the balanced interaction between ginseng and its endophytic flora is disrupted, which promotes the reproduction of *Ascomycota* within the roots. As the disease advanced, the first dominant genus of bacteria in the roots of ginseng underwent turnover as the disease worsened. The dominant genus *Pantoea* in the healthy group was replaced by *Serratia* and *Pseudomonas* in the moderately susceptible (BLS2 group) and severely susceptible (BLS4 group) groups, respectively. The *Pantoea* (*p* < 0.01) and *Pseudomonas* (*p* < 0.05) bacteriophage spp. were significantly enriched in healthy ginseng roots, and the abundance tended to decrease with the aggravation of the disease. The relative quantity of pathogenic fungi in ginseng roots from the genera *Gibberella*, *Nectria*, *Ilyonectria*, and *Alternaria* increased as the disease progressed. Furthermore, in the endophytic fungal LEfSe study, *Neonectria* was identified as the unique biomarker for the BLS3 group. The study found that fungi from the genus *Ilyonectria* were the predominant indicators for rust rot disease in American ginseng, brown rot in asparagus (Wang et al., 2023), brown rot in asparagus (Tang et al., 2022), and root rot in yellow dock (Lyu et al., 2023). Zhang et al. (Chen et al., 2023) found a significant increase in the presence of *Gibberella*, *Nectria*, and *Fusarium* in the soil between the roots of susceptible kiwifruit plants. This rise caused the development and worsening of root rot by undermining the kiwifruit’s resistance. More than 95% of *Alternaria* species are parthenogenetic parasites that can infect various plants, primarily agricultural and cash crops (Li L. et al., 2021; Qiu and Liu, 2024). The increased relative richness and enrichment of harmful fungi such as *Gibberella*, *Neonectria, Ilyonectria*, *Nectria*, and *Alternaria* are among the most significant factors contributing to root rot disease in ginseng. Therefore, after ginseng is infected with root rot, the changes in microorganisms within the roots of infected plants disrupt the original microbial antagonistic balance in the endophytic microecosystem of the plants. This destruction of the original ecological equilibrium positions of endophytic bacteria leads to a weakening of ginseng’s resistance, which in turn causes the concurrent occurrence and aggravation of the disease in ginseng.

However, the plant’s microbiome has evolved alongside it, and when pressured by root rot pathogens, ginseng will fight pathogenic bacteria with specific secretions or by recruiting good flora (Ahuja et al., 2012; Liu et al., 2017). *Rhodococcus* (*p* < 0.01), *Stenotrophomonas* (*p* < 0.05), Var*iovorax* (*p* < 0.01), and *Achromobacter* (*p* < 0.01) spp. were significantly enriched in the roots of diseased plants. These genera are documented in the literature. According to Li Z. et al. (2021), when harmful fungi are present, Astragalus mongolia recruits several beneficial bacteria to the inter- and intra-root zones, including *Stenotrophomonas*, *Achromobacter*, *Pseudomonas*, and *Flavobacterium*. The synthesis community produced by these bacteria could be enhanced by activating the immune system of plant communities, enabling plants to defend themselves against diseases by triggering systemic immunity. *Rhodococcus*, *Pantoea,* and *Serratia* genera are highly salt-tolerant and carry out biotrophic interactions, biocontrol, organic pollutant degradation, and antioxidant activity (Gu et al., 2021). As a result, these beneficial bacteria increased by diseased plants may represent a plant’s regulatory response to adversity stress, improving resilience through microbiome remodeling. However, whether these bacteria can synthesize communities to help ginseng cooperate against diseases, and the mechanism by which they help ginseng to resist diseases is not clear, and further research needs to be followed up.

### 4.3 Effect of disease occurrence on microbial function in ginseng roots

Secondary functional layer analysis of ginseng intra-root bacteria revealed that diseased ginseng had much higher levels of critical metabolic pathways such as amino acid metabolism, energy metabolism, and terpene and polyketide metabolism. Previous research has demonstrated that plants can attract beneficial microbes from a distance by actively producing non-volatile root secretions or organic compound combinations (Gao et al., 2021; Schulz-Bohm et al., 2018). Huang et al. (2022) found that 52% of Arabidopsis root-specific bacteria were involved in triterpene production. As a result, we propose that increased terpene and polyketide metabolism are critical for plant defense and signal transduction. Furthermore, diseased ginseng requires more amino acids, disease-fighting proteins, and energy for pathogen resistance and repair of injured tissues in response to disease. On the contrary, metabolic pathways such as glycan biosynthesis and metabolism, transcription, and enzyme families were significantly lower in diseased ginseng than in healthy ginseng, which adversely affects normal ginseng growth and development. The appearance of spots and white mycelium on the roots is most likely a result of this issue. Abundant symbiotic nutrient-rich fungi can effectively enhance the efficiency of plant nitrogen and phosphorus uptake (Dahlberg, 2001). They can also combat plant pathogens through antagonism, decrease the proliferation of pathogenic nutrient-rich fungi, and hinder pathogen colonization, consequently diminishing plant disease severity (Frew et al., 2018). After becoming susceptible to disease, ginseng groups showed a decrease in symbiotic nutrient-type fungi, potentially indicating a deficiency, which suggests that ginseng resistance will further deteriorate. Meanwhile, the pathotrophic phenotypes of ginseng (BLS2) rose dramatically in moderately susceptible ginseng, obtaining nutrients primarily by harming plant cells (Xiong et al., 2020). This indicates that as ginseng disease advances to stage 2, the variety of potential pathogenic fungi increases, along with the probability of pathogenicity, leading to the disturbance of the ginseng endophytic micro-ecosystem. Pathogenic-saprophytic-symbiotic nutritive fungi increased considerably in the highly susceptible groups (BLS3 and BLS4). However, they could not resist the pathogenic bacteria, which caused the ginseng endophytic microecosystem to shift from physiological equilibrium to pathological imbalance. This eventually worsened ginseng’s condition, leading to deterioration or even death.

## 5 Conclusion

This study elucidates the dynamic changes in the endophytic microbial community structure of ginseng during *F.solani*-induced root rot. The results demonstrate that persistent infection by *F. solani* significantly alters the diversity and structural composition of ginseng endophytic flora. The replacement of dominant endophytic bacterial populations and the functional shift in endophytic fungal nutrition lead to an imbalance in the diversity of ginseng endophytic microbiota, which may be a critical factor exacerbating root rot. These findings reveal a unique response pattern of ginseng endophytic microbial diversity to pathogen invasion. By exploring the microecological mechanisms underlying ginseng root rot, this study clarifies the structural changes in endophytic microbial communities under disease conditions, providing a theoretical foundation for developing effective biological control strategies.

## Data Availability

The original contributions presented in the study are publicly available. This data can be found here: NCBI SRA, accession PRJNA1167876 (bacteria) and PRJNA1167695 (fungi).

## Footnotes

^1^ https://github.com/picrust/picrust2

^2^ https://www.funguild.org/
