# Supplementary material for: Changes in the diversity of ginseng endophyte flora driven by Fusarium solani
Source: Front Microbiol. 2025 Apr 28;16:1554706. doi: 10.3389/fmicb.2025.1554706 (PMC12066479; doi:10.3389/fmicb.2025.1554706)
Supplement: Supplementary file 1 [file Data_Sheet_1.docx]

**Table S1**

**First-round PCR reaction conditions for endophytic bacteria**

| **PCR reaction temperature** | **PCR reaction time** | **cyclic number** |
| --- | --- | --- |
| 94℃ | 3 min |  |
| 94℃ | 1 min | 30 cycles |
| 70℃ | 1 min | 30 cycles |
| 54℃ | 1 min | 30 cycles |
| 72℃ | 2 min | 30 cycles |
| 72℃ | 5 min |  |
| 4℃ | ∞ |  |

**Table S2**

**Second-round PCR reaction conditions for endophytic bacteria**

| **PCR reaction temperature** | **PCR reaction time** | **cyclic number** |
| --- | --- | --- |
| 98℃ | 1 min |  |
| 98℃ | 10 s |  |
| 52℃ | 30 s | 10 cycles |
| 72℃ | 30 s |  |
| 72℃ | 10 min |  |
| 4℃ | ∞ |  |

**Table S3**

**Third-round PCR reaction conditions for endophytic bacteria**

| **PCR reaction temperature** | **PCR reaction time** | **cyclic number** |
| --- | --- | --- |
| 95℃ | 2min |  |
| 95℃ | 20 s |  |
| 52℃ | 30 s | 11cycles |
| 72℃ | 20 s |  |
| 72℃ | 10 min |  |
| 4℃ | ∞ |  |

**Table S4**

**First-round PCR reaction conditions for endophytic fungi**

| **PCR reaction temperature** | **PCR reaction time** | **cyclic number** |
| --- | --- | --- |
| 94℃ | 5 min |  |
| 94℃ | 1 min |  |
| 50℃ | 50 s | 20 cycles |
| 68℃ | 1 min |  |
| 68℃ | 10 min |  |
| 4℃ | ∞ |  |

**Table S5**

**Second-round PCR reaction conditions for endophytic fungi**

| **PCR reaction temperature** | **PCR reaction time** | **cyclic number** |
| --- | --- | --- |
| 94℃ | 1 min |  |
| 94℃ | 10 s |  |
| 50℃ | 30 s | 19cycles |
| 72℃ | 45 s |  |
| 72℃ | 10 min |  |
| 4℃ | ∞ |  |

**Table S6**

**Diversity of the 16 S rRNA gene-based bacterial and ITS rRNA gene-based fungi communities.**

|  |  | **Observed ASVs** |  | **Simpson** |  | **Coverage** |
| --- | --- | --- | --- | --- | --- | --- |
| **Bacteria** |  |  |  |  |  |  |
| HG |  | 388.2±164.65b |  | 0.878±0.123b |  | 99.99% |
| BLS1 |  | 442.4±168.22ab |  | 0.852±0.111b |  | 99.98% |
| BLS2 |  | 691.6±97.35a |  | 0.974±0.01a |  | 99.99% |
| BLS3 |  | 512.8±153.15ab |  | 0.913±0.071ab |  | 99.99% |
| BLS4 |  | 504.4±177.5ab |  | 0.911±0.111ab |  | 99.99% |
| **Fungi** |  |  |  |  |  |  |
| HG |  | 32.4±4.51a |  | 0.104±0.049a |  | 99.99% |
| BLS1 |  | 28.4±4.56a |  | 0.084±0.037a |  | 99.99% |
| BLS2 |  | 29±6.4a |  | 0.062±0.034a |  | 99.99% |
| BLS3 |  | 32.8±2.95a |  | 0.13±0.072a |  | 99.99% |
| BLS4 |  | 31±8.89a |  | 0.154±0.211a |  | 99.99% |

**Values are the means ± standard errors (n=5. Different letters in the same row mean significant difference at *P* <0.05 among the five treatments.**
